# Spatiotemporal Clustering of Middle East Respiratory Syndrome Coronavirus (MERS-CoV) Incidence in Saudi Arabia, 2012–2019

**DOI:** 10.3390/ijerph16142520

**Published:** 2019-07-15

**Authors:** Khalid Al-Ahmadi, Sabah Alahmadi, Ali Al-Zahrani

**Affiliations:** 1 King Abdulaziz City for Science and Technology, P.O. Box 6086, Riyadh 11442, Saudi Arabia; 2 King Faisal Specialist Hospital and Research Centre, P.O. Box 3354, Riyadh 11211, Saudi Arabia

**Keywords:** Middle East respiratory syndrome, coronavirus, spatiotemporal cluster, GIS, epidemiology, outbreak, Saudi Arabia

## Abstract

Middle East respiratory syndrome coronavirus (MERS-CoV) is a great public health concern globally. Although 83% of the globally confirmed cases have emerged in Saudi Arabia, the spatiotemporal clustering of MERS-CoV incidence has not been investigated. This study analysed the spatiotemporal patterns and clusters of laboratory-confirmed MERS-CoV cases reported in Saudi Arabia between June 2012 and March 2019. Temporal, seasonal, spatial and spatiotemporal cluster analyses were performed using Kulldorff’s spatial scan statistics to determine the time period and geographical areas with the highest MERS-CoV infection risk. A strongly significant temporal cluster for MERS-CoV infection risk was identified between April 5 and May 24, 2014. Most MERS-CoV infections occurred during the spring season (41.88%), with April and May showing significant seasonal clusters. Wadi Addawasir showed a high-risk spatial cluster for MERS-CoV infection. The most likely high-risk MERS-CoV annual spatiotemporal clusters were identified for a group of cities (*n* = 10) in Riyadh province between 2014 and 2016. A monthly spatiotemporal cluster included Jeddah, Makkah and Taif cities, with the most likely high-risk MERS-CoV infection cluster occurring between April and May 2014. Significant spatiotemporal clusters of MERS-CoV incidence were identified in Saudi Arabia. The findings are relevant to control the spread of the disease. This study provides preliminary risk assessments for the further investigation of the environmental risk factors associated with MERS-CoV clusters.

## 1. Introduction

Middle East respiratory syndrome coronavirus (MERS-CoV) is an emerging human viral respiratory infectious disease caused by a novel coronavirus. It was first reported in Saudi Arabia in 2012 [1], and since then, it has spread to several other countries, resulting in global public health implications. From April 2012 through February 2019, a total of 2374 laboratory-confirmed MERS-CoV cases (with 823 deaths, 34.66%) were reported to the World Health Organization (WHO) by 27 countries, with the majority (1983 cases, 83.52%) being reported by Saudi Arabia (with 745 deaths, 37.56%) [2].

The risk assessment of MERS-CoV infection, transmission and severity is crucial in predicting and preventing further outbreaks of human infections and in enhancing control measures. Recent studies have advised that dromedary camels (*Camelus dromedarius*) serve as a reservoir host for MERS-CoV, and camel-to-human transmission can occur through sporadic zoonotic infections associated with exposure to infected dromedary camels and their products [3,4,5]. The risk factors were identified for primary MERS-CoV infection in persons with either direct or indirect exposure to camels. In particular, these included persons who handle live camels (e.g., camel workers, camel salesmen, camel shepherds, slaughterhouse workers, animal and waste transporters) and persons who administer medications to camels or clean camel medical equipment [6,7,8]. Nevertheless, most patients report no exposure to camels [9]. The global camel population is estimated at approximately 35 million camels [10]; of these, 95% are dromedary camels [11]. In Saudi Arabia, the camel is considered a national, socio-cultural heritage animal and is central in the livestock economy, as it is used for milk or meat and in a traditional race festival (locally known as Mazain al-Ibal) [12]. The number of camels in Saudi Arabia is estimated at more than 1.39 million heads [13] and has been growing annually by 5.2% since 1961 [10]. Information is lacking regarding the distribution of camels in Saudi Arabia. In June 2019, the Saudi Ministry of Environment, Water and Agriculture launched an electronic system for numbering and tracking camels in order to create a database on them through the use of microchip implants [14]. This endeavor is a part of a wider initiative to control animal diseases and to create a database on each camel and its owner. The new project will help improve camel health and control the spread of zoonotic diseases.

No permanent and sustained human-to-human transmission has been observed despite close contact with MERS-CoV-infected patients [15]. However, nosocomial transmission in healthcare facilities between patients and healthcare workers and between patients sharing spaces in healthcare facilities represents the main mode of human-to-human infection [16]. MERS-CoV outbreaks linked to healthcare settings have occurred in several countries, with the largest being in Saudi Arabia, especially in 2014 and 2015 [17,18,19]. This may be due to inadequate, inconsistent or incomplete compliance with infection prevention and control practices, limited isolation of suspected MERS-CoV patients and patient crowdedness [20]. Furthermore, household transmission was observed from patients to other household members, but this was less striking than other modes of transmission [15,17,18,21]. In Saudi Arabia, the probable risk factors for MERS-CoV infection were older age [20,22], male sex [22], exposure to dromedary camels, comorbidities and smoking [23]. For household transmission, the identified risk factors were sleeping in an index patient’s room, removing a patient’s waste or touching respiratory secretions [21]. The factors related to MERS-CoV mortality were mainly old age and the existence of underlying comorbidities [22,24].

The spread of an infectious disease is an instance of spatiotemporal diffusion processes at work, in which the emergence of the disease moves across space and changes in extent over time [25]. Geographic information systems (GIS) offer a means to visualize, analyze and understand the distribution of infectious diseases over space and time, thereby revealing spatiotemporal trends, patterns and associations between the pathological factors and the geographic environments of the disease [26,27]. Exploiting the analytical, modelling and simulation features of GIS has improved epidemiological outbreak investigation and response, in which recognizing the spatial spread and transmission dynamics of an emerging infectious disease is essential for advising on efficient disease control and prevention measures [28]. However, since the first reported case of MERS-CoV in 2012, there has been a paucity of studies addressing the spatial dimension of the distribution of this disease. With the use of ecological modelling in a GIS environment [29], a preliminary transmission risk map of MERS-CoV was produced across the Middle East by analyzing the spatial distribution of MERS-CoV cases and environmental transmission risk factors (bioclimatic variables). This suggests that the transmission route of the disease via camel exposure plays a major role. However, the effects of environmental risk factors on disease transmission were not explicitly determined. A geo-additive regression model was also utilized to examine the risk factor levels of individuals for MERS-CoV mortality in the Arabian Peninsula between 2012 and 2015 [30], and significant spatial variation and clustering with a lower risk were found in Riyadh, Arar, Aljouf and Jizan, whereas a higher risk was observed in Qassim province. However, the spatial analysis lacked detail at the governorate and city levels. The application of an iterative empirical process in a GIS environment revealed that high-camel-density areas, camel convergence points (camel markets, barns, breeding sites and slaughterhouses), former MERS-CoV cases and high seroprevalence rates were the potential risk factors for spatial MERS-CoV hotspots in Kenya [31]. Considering the space and time dimensions of the MERS-CoV, one study highlighted that higher heterogeneity was more prominent in zoonotic than in human-to-human transmission in the Middle East region; this result emphasizes the importance of the environmental component of the epidemic [32].

Although approximately 83% of the globally reported MERS-CoV cases are found in Saudi Arabia, spatial patterns and clusters of the occurrence of this disease have not been addressed, leaving a wide gap in knowledge on this important issue. This study aimed to examine the spatiotemporal clustering of the MERS-CoV incidence in Saudi Arabia between 2012 and 2019 using spatial scan statistics and GIS.

## 2. Materials and Methods

### 2.1. MERS-CoV Data

All laboratory-confirmed MERS-CoV cases reported between June 13, 2012 and March 31, 2019 were compiled from the official websites of the Saudi Ministry of Health (SMOH) [33] and the WHO [34]. We undertook a detailed review of the MERS-CoV data and performed a range of checks for data consistency, completeness and fitness for the study purpose. We then developed a dataset with variables of interest for each individual with MERS-CoV. The dataset included the following: diagnosis date, gender, age, nationality, healthcare, employment status and source of infection, as well as the city, governorate and province of residence.

A confirmed case is defined as a suspected case that has a laboratory confirmation of MERS-CoV infection. A suspected case is defined as either (i) an adult patient presenting with severe pneumonia or acute respiratory distress syndrome, based on clinical or radiological evidence, or (ii) an adult patient presenting with an unexplained deterioration of a chronic condition, such as congestive heart failure or chronic kidney disease being treated with hemodialysis, or (iii) a child or an adult patient exposed to a confirmed case of MERS-CoV or who has visited a healthcare facility where a MERS-CoV patient was recently identified, or has had a history of contact with dromedary camels or consumption of camel products within 14 days before symptoms and who presents with either (a) acute febrile illness (temperature ≥ 38 °C) with or without respiratory symptoms, or (b) gastrointestinal symptoms and leukopenia or thrombocytopenia. Laboratory testing for MERS-CoV is performed at approved regional SMOH and selected non-SMOH governmental laboratories to confirm a clinically suspected case and to screen contacts by using validated, commercial, real-time, reverse-transcription polymerase chain reaction (rRT-PCR) assays. The laboratory confirmation of MERS-CoV infection requires either a positive rRT-PCR result for at least two specific genomic targets, or a region upstream and open reading frame1a (upE and ORF1a) [35].

A primary case is defined as a person with a laboratory-confirmed MERS-CoV infection with no evidence of contact with infected individuals but is known or believed to have had direct or indirect exposure to camels or camel habitats. Exposure to camels includes direct physical contact with camels or their surroundings (milking and handling excreta), drinking raw camel milk or other unpasteurized products derived from camel milk and handling raw camel meat. Indirect contact includes casual contact with sites where camels have been (e.g., camel markets or farms) but without direct physical contact with camels, or living with a household member who has had direct contact with camels. By contrast, a secondary case is defined as a person who has shared the same enclosed space (e.g., a room or office) for frequent or extended periods with an individual with a symptomatic MERS-CoV infection. MERS-CoV is believed to spread between humans mainly through contact and respiratory droplets. However, transmission through small particle droplet nuclei (aerosols) may occur. Environmental contamination during outbreaks in healthcare facilities can be extensive and might contribute to outbreak amplification, if adequate disinfection procedures are not followed [35].

### 2.2. Spatial Data

The spatial database of the MERS-CoV incidence in Saudi Arabia was created in the format of an ESRI file geodatabase on the three spatial levels of city, governorate and province. Saudi Arabia consists of 13 administrative provinces, 136 governorates and more than 300 cities. MERS-CoV cases were grouped and aggregated to be represented by cities, governorates and provinces.

### 2.3. Empirical Bayes (EB) Smoothed Rate Maps

The prevalence of intrinsic variance instability in estimating incidence rates as a result of the variation in populations across spatial units, which can possibly identify outliers, has received broad attention in the disease mapping field [36]. To address this issue, we used GeoDa [37] software for generating EB smoothed rate maps for MERS-CoV incidence. The number of MERS-CoV incidence cases for the governorates was used as an event variable, and the populations of governorates were estimated from the 2010 census [38] and used as base variables.

### 2.4. Spatial Cluster Analysis

We analyzed the spatiotemporal clustering of the MERS-CoV incidence in Saudi Arabia between 2012 and 2019 at the city level by using Kulldorff’s spatial scan statistics via SaTScan 9.6 [39]. We used purely temporal, seasonal, purely spatial and spatiotemporal retrospective analyses to scan, detect and evaluate the periods and geographical areas with the highest MERS-CoV risk incidence clusters. The purely spatial scan statistic is defined by a circular window on the map. The window is sequentially centered on each of several possible cities that are positioned throughout the study area. The spatiotemporal scan statistic imposes a cylindrical window with a circular geographic base and height corresponding to time. The temporal scan statistic uses a window that moves in one dimension, time, defined in the same way as the height of the cylinder used by the spatiotemporal scan statistic. The key feature that distinguishes the seasonal scan statistic from the purely temporal scan statistic is that the former ignores the year of the observation and retains only the day and month [40]. The number of MERS-CoV cases by city was used as the case file, the city population estimated from the 2010 census [38] was used as the population file and the latitude and longitude of each city were used in the coordinates file.

In SaTScan, an analysis was conducted by progressively scanning a window across time and/or space through a comparison of the number of observed and expected cases of MERS-CoV incidence, assuming random distribution, inside the window at each city. The null hypothesis is that the incidence of MERS-CoV is randomly distributed, and the alternative hypothesis is that the incidence increases more inside the window than in areas outside it. The log likelihood ratio (LLR) is the hypothesis-testing statistic estimated based on Monte Carlo randomization. The window with the maximum likelihood ratio is the most likely cluster; that is, it identifies the cluster that is least likely to occur by chance. In addition to the most likely cluster, SaTScan also designates secondary clusters for purely spatial and spatiotemporal analyses and ranks them in relation to their estimated LLR statistic. SaTScan scans for clusters by using different criteria; the criterion recommended by SaTScan is the percentage of the population at risk, with a value of 50% [40]. We tested the percentage of the population at risk from 10% to 50%, and from the result, 30% performed best; that is, the value of 30% did not include neighboring cities that have a non-elevated risk. The four types of analyses (purely temporal, seasonal, purely spatial and spatiotemporal) were conducted using the Poisson discrete-based model with 999 Monte Carlo permutations to test for statistical significance. Only clusters with significance levels of 0.05 and only scans of cities with high rates were reported. For temporal analysis, values of 1 day, 1 month and 1 year were set as the time aggregation units for the daily, monthly and annual clusters, respectively, whereas for the seasonal cluster, 1 month was set. For spatiotemporal analysis, 1 month and 1 year were set for the monthly and annual spatiotemporal analyses, respectively.

## 3. Results

### 3.1. Overall

A total of 2008 laboratory-confirmed human MERS-CoV cases reported in Saudi Arabia during the period between June 2, 2012 and March 31, 2019 were included in this study. The primary cases accounted for 24.05% (*n* = 483) of the total number of confirmed cases; of these, 48.24% (*n* = 233) involved (direct and indirect) exposure to camels. Secondary cases accounted for 40.90% (*n* = 821) of the total number of confirmed cases, whereas missing and unknown cases accounted for 18.02% (*n* = 362) and 17.03% (*n* = 342) of the total number of confirmed cases, respectively.

### 3.2. Temporal Trend of MERS-CoV Infection

**Figure 1:**
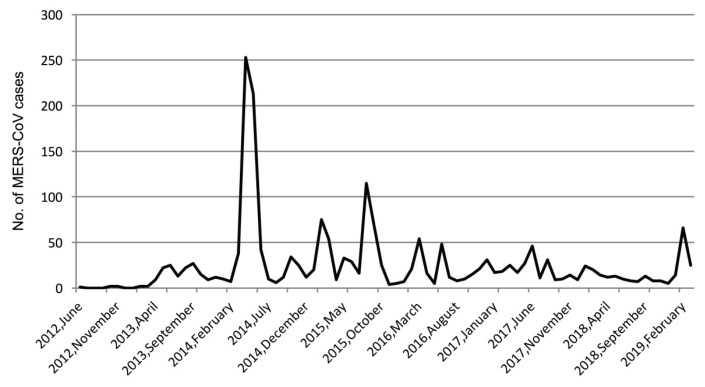
Epidemic curve of Middle East respiratory syndrome coronavirus (MERS-CoV) incidence in Saudi Arabia between 2012 and 2019.

On an annual basis, most of the MERS-CoV cases in Saudi Arabia between 2012 and 2019 occurred in 2014 (*n* = 662, 32.97%), followed by 2015 (*n* = 454, 22.61%). Afterwards, the MERS-CoV incidence decreased between 2016 and 2019 (*n* = 105 to 248, 5.23% to 12.35%). In terms of the seasonality of MERS-CoV infections, our results showed that the largest proportion of total MERS-CoV infections occurred during spring (*n* = 841, 41.88%). This was followed by summer (*n* = 460, 22.91%), winter (*n* = 375, 18.68%) and fall (*n* = 332, 16.53%). On a monthly basis, approximately one-third (31.33%) of the total MERS-CoV cases occurred during April (*n* = 316) and May (*n* = 313), followed by March, February and June (*n* = 201 to 212; 10.01% to 10.56%). The epidemic curve of MERS-CoV infection in Saudi Arabia during the seven-year study period showed significant variations over time (Figure 1). Two prominent outbreaks of MERS-CoV infection were observed in 2014, one in April (*n* = 253, 12.59%) and another in May (*n* = 213, 10.60%). This was followed by an outbreak in August 2015 (*n* = 115, 5.72%). Two minor outbreaks with fairly comparable numbers of MERS-CoV cases (*n* = 69 and *n* = 66) occurred in September 2015 and February 2019. In addition to the observed epidemics, sporadic MERS-CoV cases were also reported throughout the study period.

### 3.3. Spatial Pattern of MERS-CoV Infection

**Figure 2:**
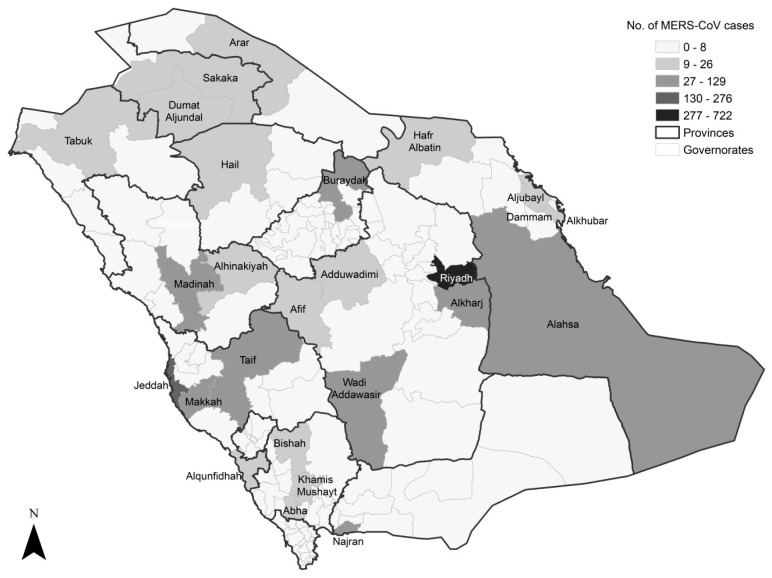
Spatial distribution of the number of MERS-CoV cases in Saudi Arabia between 2012 and 2019. The presented data are for the governorate level, with the provincial boundaries overlaid.

**Figure 3:**
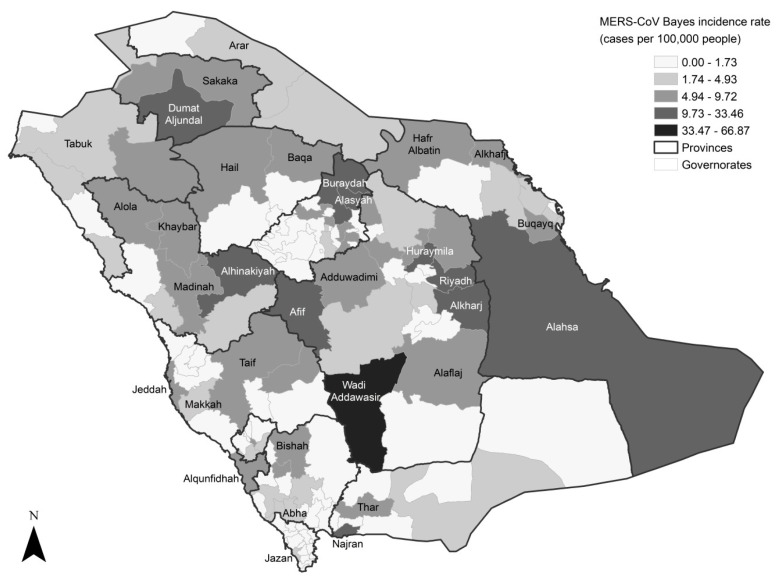
Empirical Bayes smoothed incidence rate of MERS-CoV in Saudi Arabia between 2012 and 2019. The presented data are for the governorate level, with the provincial boundaries overlaid.

The incidence of MERS-CoV infection was mostly reported from Riyadh (*n* = 722, 35.95%), Jeddah (*n* = 276, 13.74%) and Alahsa governorates (*n* = 129, 6.42%), Figure 2. The incidence in Wadi Addawasir, Buraydah, Taif, Alkharj, Najran, Madinah and Makkah governorates ranged from 51 to 81 cases. These were followed by five governorates in northern Saudi Arabia (Hafr Albatin, Sakaka, Hail, Dumat Aljundal and Tabuk) and two governorates in eastern Saudi Arabia (Alkhubar and Dammam) with 20–26 cases. For the EB smoothed incidence rate of MERS-CoV infection, the Wadi Addawasir governorate showed the highest rate across the country, with 66.87 cases per 100,000 people (Figure 3). Dumat Aljundal and Najran governorates followed with 33.46 and 20.02 cases per 100,000 people, respectively. Alkharj, Alhinakiyah and Afif governorates exhibited an incidence rate in the range of 15.03–18.12 cases per 100,000 people. In Riyadh, the capital of Saudi Arabia, the incidence rate of MERS-CoV infection was 13.92 cases per 100,000 people. In Buraydah, Alahsa and Jeddah governorates, the incidence rates were 13.04, 12.06 and 7.98 cases per 100,000 people, respectively.

### 3.4. Spatiotemporal Clustering of MERS-CoV Infection

Temporal cluster analysis generated from the spatial scan test identified the years 2014, 2015 and 2016, the months of April and May of 2014, and the period from April 5 to May 24, 2014 as the strongly significant clusters of annual, monthly and daily MERS-CoV incidence, respectively (Table 1). Seasonal cluster analysis revealed that April and May show strongly significant seasonal clusters of MERS-CoV incidence (Table 1).

**Figure 4:**
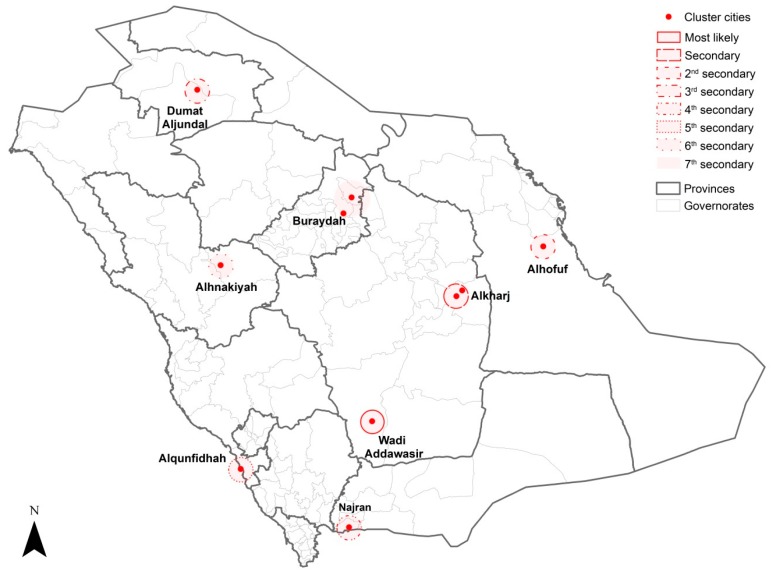
Locations of the spatial clusters of MERS-CoV incidence in Saudi Arabia between 2012 and 2019.

The results of the purely spatial cluster analysis of MERS-CoV incidence from 2012 to 2019 revealed the most significant and secondary clusters at the city level (Table 2 and Figure 4). Wadi Addawasir in Riyadh province had the most likely high-risk cluster, followed by a secondary significant cluster in Alkharj and Aldilm cities in the same province. Spatial clusters in single cities were identified across the country; Alhofuf (east), Dumat Aljundal (north), Najran (south), Alqunfidhah (southwest), Alhinakiyah (west) and Buraydah (center) represented the third, fourth, fifth, sixth, seventh and eighth secondary clusters, respectively.

**Figure 5:**
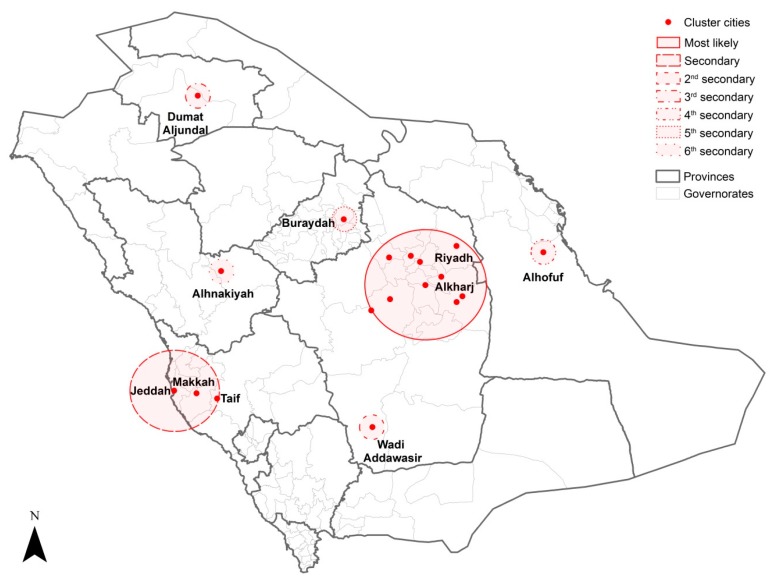
Locations of the annual spatiotemporal clusters of MERS-CoV incidence in Saudi Arabia between 2012 and 2019.

**Figure 6:**
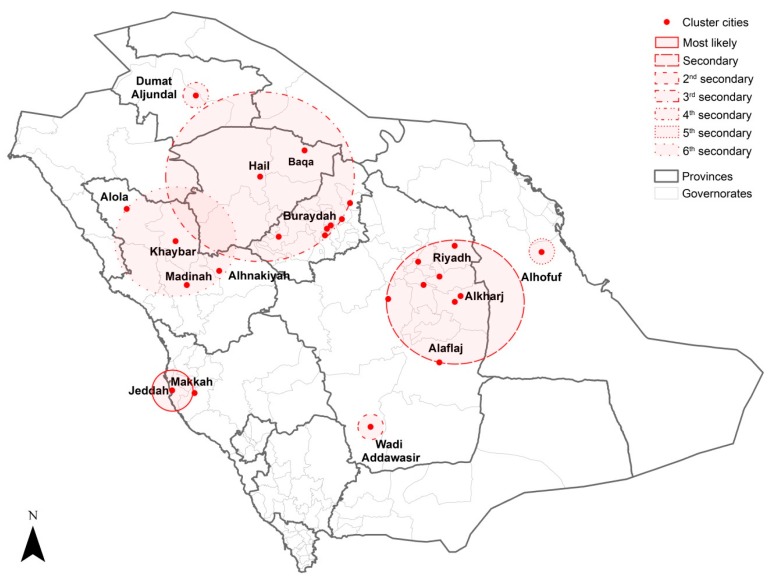
Locations of the monthly spatiotemporal clusters of MERS-CoV incidence in Saudi Arabia between 2012 and 2019.

The results of the spatiotemporal cluster analysis of MERS-CoV infection, using years and months as the time aggregates from 2012 to 2019, showed significant most likely and secondary clusters in Saudi Arabia (Table 3; Table 4 and Figure 5; Figure 6). Spatial variations existed between the annual and monthly spatiotemporal clusters. For the annual spatiotemporal clusters, a group of cities (*n* = 10) located in Riyadh province was identified as the most likely high-risk cluster for MERS-CoV incidence between 2014 and 2016. This was followed by a secondary cluster that was found for three cities (Jeddah, Makkah and Taif) in Makkah Province between 2014 and 2015. Spatiotemporal clusters in single cities were also observed and varied in space and time across the country. Wadi Addawasir (2018–2019), Dumat Aljundal (2017–2018), Alhofuf (2015–2016), Buraydah (2015–2016) and Alhinakiyah (2014–2015) represented the second, third, fourth, fifth and sixth secondary clusters, respectively.

In contrast to the annual cluster analysis, the monthly spatiotemporal cluster analysis identified Jeddah, Makkah and Taif cities as the most likely high-risk cluster for MERS-CoV incidence between April and May of 2014. In addition, a group of cities (*n* = 7) located in Riyadh province was identified as a secondary cluster between March 2014 and October 2015. In the most recent MERS-CoV outbreaks, a single spatiotemporal second secondary cluster of MERS-CoV cases was identified in Wadi Addawasir in February 2019. A cluster (third secondary cluster) of eight cities in the central-north of Saudi Arabia located in Qassim and Hail provinces was observed in March 2016. Clusters in Dumat Aljundal in August 2017 and Alhofuf in May and June 2015 were identified as the fourth and fifth secondary clusters, respectively. Moreover, a cluster that encompassed several cities (*n* = 4) located in Madinah province in western Saudi Arabia was identified as the sixth secondary cluster between April and June 2014.

## 4. Discussion

In this study, we examined the spatial pattern of MERS-CoV risk at the governorate level and the temporal, seasonal, spatial and spatiotemporal clustering of MERS-CoV incidence at the city level over a seven-year period. To the authors’ knowledge, this is the first study that aims to analyze the spatiotemporal pattern and clustering of MERS-CoV in Saudi Arabia. A total of 2008 laboratory-confirmed MERS-CoV cases were reported in Saudi Arabia, representing approximately 83% of the global cases. Overall, the majority of MERS-CoV cases were secondary infections (40.90%). This result indicates that secondary infections, either hospital or community acquired, remain a major challenge for the Saudi healthcare system in the prevention and control of MERS-CoV outbreaks, despite the significant improvement in MERS-CoV surveillance. On the other hand, the primary cases accounted for only 24.05% of the total confirmed cases. Although compared with the general population, people in close contact with dromedary camels have a higher risk of developing MERS-CoV infection via a primary source [3,4,5], our results indicated that only 48.24% of the total primarily infected cases were associated with direct or indirect contact with camels. This result is consistent with previous findings [4] regarding the ambiguity of primary MERS-CoV infection transmission. In Saudi Arabia, camel milk and meat production has increased by 5.4% and 6.4% per year, respectively [41]. However, intensified animal production has epidemiological consequences, including increased risk of disease. Recent trends in Saudi Arabia have indicated a tendency towards dromedary camel husbandry intensification since the 1960s, as evident in increased production in nearby cities by providing enhanced supplemental diets for the animals and improving camel health management [11,42,43]. These areas of intensified camel production are probable hotspots for the transmission and spread of MERS-CoV. In addition, dealing with camel products, consuming raw unpasteurized milk and conducting slaughter processes have been documented as risk factors for primary MERS-CoV transmission to humans [44].

The epidemic curve of MERS-CoV has varied significantly each year from 2012 to 2019, and it has exhibited variance in monthly peaks. A combination of sporadic and epidemic patterns as a result of animal-to-human, human-to-human and unknown exposure was observed. By contrast, the epidemic curve in South Korea, where the largest outbreak outside of the Arabian Peninsula occurred, had a clear nosocomial epidemiological pattern [45]. Purely temporal cluster analyses of MERS-CoV infection illustrated significant clusters in April and May of 2014. This finding is consistent with previous results [46], which showed significant peaks in MERS-CoV incidence between March and May during a similar period. Seasonal cluster analysis identified April and May as a strongly significant seasonal cluster of MERS-CoV infection. In accordance with our findings, it was reported in [47] that MERS-CoV infection occurred markedly in June, followed by May and April, and the lowest rates were seen in January. One possible reason for this trend is the seasonal variations in zoonotic infections in camels during the breeding season [48,49], when camel farms are considered an important potential source of MERS-CoV transmission [47]. Moreover, a recent serological study in Saudi Arabia found a higher risk of MERS-CoV infection in camels in winter than in summer [50]. However, the low daily frequency and sporadic cases of MERS-CoV indicate a reduced likelihood of zoonotic-to-human transmission and increased possibility of human-to-human transmission, which is consistent with other findings [51]. The main MERS-CoV outbreaks in 2014 and 2015 were closely followed by human influenza A epidemics [52]. No coincidence was found between the peak of influenza occurrence and MERS-CoV occurrence, which suggests that the seasonal characteristics of MERS-CoV infections may vary from those of human influenza viral infections. In this study, no epidemics of MERS-CoV were observed during mass gatherings of pilgrims in the Hajj season, which is consistent with previous findings [53]. This indicates another knowledge gap regarding the mode of transmission that needs further investigation.

Spatial clusters of MERS-CoV cases were mainly found in a group of cities in the provinces of Riyadh, Qassim, Hail and Najran located on the outskirts of larger deserts, which is the natural habitat for camels. However, significant spatial clusters of MERS-CoV cases were also reported from small general hospitals in single cities, such as Wadi Addawasir, Dumat Aljundal, Alhinakiyah and Alqunfidhah; in these settings, delays in the isolation of suspected patients, inadequate infection and control measures, and late case diagnosis and management are expected. The annual spatiotemporal high-risk MERS-CoV clusters occurred mainly in the early periods of the MERS-CoV epidemic (between 2014 and 2016) in major cities, such as Riyadh, Jeddah, Taif, Alhofuf and Buraydah, whereas recent clusters (between 2017 and 2019) were observed in relatively small cities, such as Dumat Aljundal and Wadi Addawasir. This result can be explained by infection prevention and control practices, which were, to some extent, more effective in major cities than in small cities and remote areas.

For the monthly spatiotemporal high-risk MERS-CoV clusters, the cities of Jeddah, Makkah and Taif were identified as a part of the most likely high-risk MERS-CoV cluster for April and May of 2014, when the number of cases represented the largest accumulation of cases reported since the beginning of the MERS-CoV outbreak. The probable source of infection in the majority of the cases in this outbreak was secondary human-to-human transmission in Jeddah that took place in healthcare facilities as a result of overcrowding and inadequate infection control measures, rather than a sudden increase in primary cases in the community [49]. The spatiotemporal cluster detected in Riyadh between March 2014 and October 2015 could be attributed to several outbreaks, with the most prominent one occurring in a single healthcare setting in Riyadh in August 2015 [54]. In addition, the most recent clusters of MERS-CoV incidence were identified in Wadi Addawasir in February 2019, in Dumat Aljundal in August 2017 and in Buraydah in March 2016; according to [55,56,57], the majority of the cases were associated with healthcare-acquired infections. This indicates persistent challenges related to nosocomial transmission, which require a thorough investigation of compliance to infection control measures by healthcare workers.

## 5. Conclusions

MERS-CoV infection has global public health implications and has been labelled as an epidemic in Saudi Arabia. This study examined the spatial pattern and spatiotemporal clusters of MERS-CoV incidence in Saudi Arabia for the first time by using the latest publicly available MERS-CoV data. The results of this study provide initial risk assessments that can be used as the basis for the further investigation of the potential environmental risk factors that can explain the spatiotemporal clusters of MERS-CoV infection. The immediate isolation of suspected patients, adequate infection control measures and early case diagnosis and management remain the principal elements in controlling the spread of the disease, especially in small hospitals and in remote areas. Future research investigating the effects of age, gender and occupation on MERS-CoV infection and mortality is needed.

## Figures and Tables

**Table 1:** Temporal and seasonal clusters of MERS-CoV incidence in Saudi Arabia between 2012 and 2019. LLR: log likelihood ratio.

Timeframe	Observed Cases	Expected Cases	LLR	Relative Risk	*p*-Value
2014 to 2016	1364	592	320	3.12	0.001
April 2014 to May 2014	466	49	677	11.98	0.001
April 5, 2014 to May 24, 2014	446	41	709	13.87	0.001
April to May	629	296	161	2.54	0.001

**Table 2:** Spatial clusters of MERS-CoV incidence in Saudi Arabia between 2012 and 2019.

Cluster Type	No. of Cities in the Cluster	Observed Cases	Expected Cases	LLR	Relative Risk	*p*-Value
Most likely	1	81	9	104	8.92	<0.001
Secondary	2	70	15	55	4.92	<0.001
Second secondary	1	128	67	23	1.97	<0.001
Third secondary	1	21	3	21	6.42	<0.001
Fourth secondary	1	68	30	18	2.30	<0.001
Fifth secondary	1	14	2	13	5.67	<0.001
Sixth secondary	1	11	2	11	6.52	<0.001
Seventh secondary	2	85	50	10	1.73	<0.001

**Table 3:** Annual spatiotemporal clusters of MERS-CoV in Saudi Arabia between 2012 and 2019.

Cluster Type	Timeframe (year)	No. of Cities in the Cluster	Observed Cases	Expected Cases	LLR	Relative Risk	*p*-Value
Most likely	2014 to 2016	10	491	163	245	3.67	<0.001
Secondary	2014 to 2015	3	315	84	201	4.28	<0.001
Second secondary	2018 to 2019	1	56	1	153	41.57	<0.001
Third secondary	2017 to 2018	1	21	0	59	43.70	<0.001
Fourth secondary	2015 to 2016	1	50	10	41	5.17	<0.001
Fifth secondary	2015 to 2016	1	33	7	25	4.79	<0.001
Sixth secondary	2014 to 2015	1	9	0	23	36.28	<0.001

**Table 4:** Monthly spatiotemporal clusters of MERS-CoV in Saudi Arabia between 2012 and 2019.

Cluster Type	Timeframe (month/year)	No. of Cities in the Cluster	Observed Cases	Expected Cases	LLR	Relative Risk	*p*-Value
Most likely	04/2014 to 05/2015	2	252	13	532	22.83	<0.001
Secondary	03/2014 to 10/2015	8	500	135	326	4.58	<0.001
Second secondary	02/2019	1	47	0	240	452.96	<0.001
Third secondary	03/2016	8	39	1	98	32.90	<0.001
Fourth secondary	08/2017	1	19	0	98	465.20	<0.001
Fifth secondary	05/2015 to 06/2015	1	38	2	83	23.54	<0.001
Sixth secondary	04/2014 to 06/2014	4	38	4	49	8.94	<0.001
